# Cognitive Behavior Therapy for Depression From an Evolutionary Perspective

**DOI:** 10.3389/fpsyt.2021.667592

**Published:** 2021-07-05

**Authors:** Steven D. Hollon, Paul W. Andrews, J. Anderson Thomson

**Affiliations:** ^1^Department of Psychology, Vanderbilt University, Nashville, TN, United States; ^2^Department of Psychology, Neuroscience and Behaviour, McMaster University, Hamilton, ON, Canada; ^3^Counseling and Psychological Services, Student Health, and Institute of Law, Psychiatry, and Public Policy, University of Virginia, Charlottesville, VA, United States

**Keywords:** depression, evolution, rumination, cognitive behavior therapy, antidepressant medications

## Abstract

Evolutionary medicine attempts to solve a problem with which traditional medicine has struggled historically; how do we distinguish between diseased states and “healthy” responses to disease states? Fever and diarrhea represent classic examples of evolved adaptations that increase the likelihood of survival in response to the presence of pathogens in the body. Whereas, the severe mental disorders like psychotic mania or the schizophrenias may involve true “disease” states best treated pharmacologically, most non-psychotic “disorders” that revolve around negative affects like depression or anxiety are likely adaptations that evolved to serve a function that increased inclusive fitness in our ancestral past. What this likely means is that the proximal mechanisms underlying the non-psychotic “disorders” are “species typical” and neither diseases nor disorders. Rather, they are coordinated “whole body” responses that prepare the individual to respond in a maximally functional fashion to the variety of different challenges that our ancestors faced. A case can be made that depression evolved to facilitate a deliberate cognitive style (rumination) in response to complex (often social) problems. What this further suggests is that those interventions that best facilitate the functions that those adaptations evolved to serve (such as rumination) are likely to be preferred over those like medications that simply anesthetize the distress. We consider the mechanisms that evolved to generate depression and the processes utilized in cognitive behavior therapy to facilitate those functions from an adaptationist evolutionary perspective.

## Introduction

In the early 1900's Emil Kraepelin, widely considered the father of modern psychiatry, stated, “it is almost impossible to establish a fundamental distinction between the normal and the morbid mental state (p. 115) (1).” Over a century later, the latest edition of the American Psychiatric Association's Diagnostic and Statistics Manual (DSM-5) meekly echoed Kraepelin's statement: “[I]n the absence of clear biological markers or clinically useful measurements of severity for many mental disorders, it has not been possible to completely separate normal and pathological symptom expressions contained in diagnostic criteria (p. 21) (2).” A century of stagnation on such a fundamental issue is an alarming lack of progress for any scientific field and it speaks to a failure to rigorously adhere to a hypothesis disconfirmation approach (3).

In particular, there has been significant debate as to whether modern diagnostic criteria for depression accurately distinguish between normal and pathological states. Not all unpleasant reactions or experiences are necessarily diseases or disorders. Fevers and diarrhea are unpleasant to experience, but they are not diseases in and of themselves; rather, they represent a coordinated effort to rid the body of dangerous pathogens (in the body generally in the case of fever, and in the gut specifically in the case of diarrhea). Unless they become too pronounced (too high a fever can produce brain damage in infants although that is rare, and people do die of dehydration in the event of protracted diarrhea) they increase the chances of survival for those who are “afflicted.”

A similar case can be made that those non-psychotic psychiatric “disorders” that are marked by strong states of negative affect (depression and anxiety especially) represent adaptations that evolved to serve a function in our ancestral past. In that sense they are neither “diseases” nor “disorders” but instead coordinated responses to external challenges or threats that increase the chances of passing on one's gene line (inclusive fitness). They may be distressing to experience and even disrupt life at times, but if they increased reproductive fitness they would have been selected by evolutionary pressures. It should be noted that evolution selects for the “*survival of the fittest gene line*” and not the “*fittest individual.”* Inclusive fitness is the sum of the reproductive fitness of the individual (direct fitness) and his or her biological relatives (indirect fitness). There are instances in which acting in ways that lessens the odds that the individual will reproduce increases the odds that his or her gene line will reproduce.

Clinicians often refer to behaviors that do not serve the individual as being “maladaptive” without recognizing that if a trait advanced the inclusive fitness of his or her ancestor's gene line in their evolutionary past then it would have been selected for by evolution and psychological mechanisms “baked in” that are there to be expressed in modern life. That will have implications for the ways in which terms like “maladaptive” are used; from an evolutionary perspective “maladaptive” means that the trait reduces inclusive fitness, whereas from a clinical perspective “maladaptive” implies that the trait is not helpful to the individual. The key point is that evolution may have selected for certain traits that are adaptive for the propagation of one's gene line but that are not propitious for the individual him or herself. Think of risk-taking and the men who live fast and die young, but leave offspring with the women who were attracted to them.

The fact that a trait evolved in our ancestral past does not mean that we necessarily have to adhere to it today if it does not suite our current purposes (most reproductively capable adults practice birth control at times), but it does facilitate the therapeutic process for the clinician to recognize and discuss with the patient that some behaviors that seem “maladaptive” may have been selected for in our ancestral past. This is a point to which we will return later in the article.

Psychiatry, especially through the DSM and psychopharmacology, has had an outsized influence on the understanding, research investigation, categorization, and treatment of mental illnesses. That is being challenged, especially with the use of an evolutionary approach. The non-psychotic disorders may be aversive to experience but motivate adaptive defenses that serve to propagate the gene line (4). It is the gene line that is selected via evolution, not the individual.

Retrospective epidemiological studies estimate that 16% of all people will experience an episode that meets modern criteria for major depression at some time in their lives (5), whereas cohort studies that follow people from birth on put that number three times as high, with the majority of those extra instances coming in response to major life stressors among people who are unlikely to experience subsequent episodes (6). Prevalence rates of that magnitude raise concerns that the diagnostic criteria for major depressive disorder are inaccurate and overinclusive (7, 8). Women are twice as likely as men to experience episodes of depression, a disparity that first emerges in early adolescence and that is maintained across the lifespan (9). That is an unusual time course for a “true” disease to follow; most kill you in your infancy or your dotage (10).

The decision to eliminate the bereavement exclusion from DSM-5 generated considerable controversy precisely because grief is widely recognized to be a “normal” response to the loss of a loved one (11–13). Almost anyone will experience a grief reaction following such a loss and that grief is largely homologous with depression. High prevalence and near universality in response to loss suggest that depression is “species typical” (something that can happen to anyone) and its gender disparity (women are twice as likely to get depressed as men), and age of onset (half of all first episodes occur in the teens) suggests that it evolved to solve life challenges relevant for young women as they enter their reproductive years (14). Women cannot “muscle” their way out of stressful situations and grip strength is inversely correlated with risk for depression (15).

In this article, we explore the implications of depression as an evolved adaptation and consider the proposition that aspects of cognitive behavior therapy (CBT) may be particularly well-suited to advance the functions that depression evolved to serve. We focus especially on the notion that melancholic depression (and perhaps most other clinical depressions as well) evolved to facilitate the process of analytical rumination (the careful consideration of the causes of and possible solutions to) complex social problems that are particularly likely to arise as young primates first take on adult responsibilities (16). “Depression” is a catchall term that encompasses multiple, evolutionarily related phenotypes (including sickness depression, starvation depression, and clinical melancholia) that share sadness and anhedonia in common, as well as some genes and neurocircuitry, but that differ in other symptoms and the situations that trigger them.

There are reasons both anatomical and biological (see below) to prefer the analytical rumination hypothesis (ARH) to other possible evolutionary explanations for melancholia (17) and other phenotypes characterized by rumination like atypical depression (18, 19) and reasons to prefer CBT or other related psychosocial interventions (also described below) to antidepressant medications (ADMs) to the extent that those anatomical and biological implications are true (20).

It is *a basic principle of evolutionary medicine that any intervention that facilitates the functions that a negative affect evolved to serve is more likely to be successful in the long run than one that merely anesthetizes the distress*. We think that CBT facilitates the functions that depression evolved to serve (it makes rumination more efficient) whereas ADMs only suppress the distress and leaves the problem that triggered the depression largely unaddressed.

To illustrate how CBT may work within the context of the ARH, we raise nine questions likely to be of interest to clinicians, and we discuss how our evolutionary approach provides insight into each. We have addressed these questions in greater detail elsewhere, and we refer interested readers to our previous articles for more in-depth considerations (16, 17, 21, 22).

## Question 1: Why Do People Have Painful Feelings? It Is All About the Squids and the Sea Bass

Most evolutionary accounts of aversive feelings propose that they are triggered by harmful events and that they motivate behavior and learning that promote avoidance of those events. Anger motivates avoidance of social exploitation (23), anxiety motivates avoidance of an imminent threat (24), jealousy motivates avoidance of romantic infidelities (25), and pain motivates avoidance of damage to bodily tissues (26). It is also commonly thought that emotional adaptations produce coordinated whole-body responses to meet the various adaptive challenges of the different situations (27–29). Even though negative emotions share a functional commonality in that all are thought to promote avoidant behavior and learning, the precise whole-body response that is triggered depends on the specific harm to be avoided. Avoiding a predator requires a different whole-body response than avoiding infidelity, a pathogen, or social ostracism.

In each instance, the external challenge is different, and the body is readied to respond in a different fashion to each so as to maximize inclusive fitness. From the perspective of evolutionary biology, such syndromes are neither diseases nor disorders. Unlike a disease, the physical structure of the body is intact and doing what it was shaped by natural selection to do. The affects that emerge coordinate a “whole body response” (thought, feeling, physiology, and behavior) that is anything but disordered.

Low prevalence, high heritability disorders like the serious mental illness (SMIs) (schizophrenia, bipolar I, and autism) may well represent “true” diseases in the classic sense of the term, but the high prevalence modestly heritable non-psychotic “disorders” that revolve around distressing affects like depression and anxiety likely represent adaptations that evolved in our ancestral past because they enhanced reproductive fitness (30).

We illustrate this point with a study on the adaptive value of physical pain. Sea bass eat squid and, as best as we can tell, squid prefer not to be eaten. Crook and colleagues conducted an elegant trial to evaluate the survival value of pain (31). In that study, quartets of squids either had a swimmer surgically removed (or not) under anesthesia (or not) in a 2x2 factorial design and were then placed in a tank with a hungry sea bass 6 hrs later (long enough for the effects of the anesthetic to wear off) with rates of predation monitored. Human observers could not detect which of the squids had been operated on, but the sea bass could (that is the kind of thing that predators evolved to do). The squids that were physically intact were the least likely to be eaten (whether they had been anesthetized or not), whereas the squids that had been operated on under anesthesia were the most likely to be eaten, largely because they began evasive maneuvers no sooner than the squids that were intact. Those squids that had been operated on without anesthesia began evading the sea bass sooner than the squids that were intact and were more successful in avoiding predation than the squids that had been maimed but felt no pain. The moral of the story is that pain may hurt, but it motivates the organism to avoid further harm and facilitates survival.

Melancholic depression is distressing, but that is not necessarily bad. If it is a normal emotional adaptation, the issue is to figure out what negative circumstances it evolved to avoid.

## Question 2: What Is the Evidence that Melancholia Is an Adaptation?

Demonstrating that a trait such as melancholia is an adaptation is an onerous burden, and we refer readers to our other papers for more thorough treatments (21, 32, 33). Natural selection is the only known force in nature capable of producing highly organized and coordinated traits, and the only workable explanation for the architecture of the brain (34, 35). As a consequence, the search for adaptation essentially involves recognizing highly organized and coordinated traits.

When faced with a trait with unknown evolutionary origins, such as melancholia, the researcher should engage in a two-step reverse engineering process. The first step involves identifying as many features of the trait as possible, including neurological and physiological components, but also cognitions, feelings, and behaviors. The second step involves attempting to identify an effect that non-randomly organizes the features. Vision non-randomly organizes all the features of the eye (cornea, lens, pupil, iris, trabecular meshwork, vitreous humor, retina, etc.), and so vision is the evolved function of the eye. A systematic failure to find evidence of organization or coordination increases confidence that other explanations are required.

As we have argued in detail elsewhere, the classic description of melancholic depression exhibits a high degree of order and coordination for promoting Type 2 avoidant learning in response to serious failures or mistakes (21). By “avoidant learning” we mean that melancholia is an emotional response to serious missteps, and that it promotes a learning style whose function is to avoid similar events in the future. By “Type 2” we refer to one of two basic information processing styles that are widely studied in cognitive psychology. Type 1 processing is quick and requires little more than instinct or a conditioned stimulus-response. That is the kind of thinking that leads one to assume that the rustle in the bushes is a predator intent on a meal and not something more benign or tasty. The premium in such instances is on rapidity of response and little time is spent in careful contemplation. In contrast, Type 2 thinking is more contemplative and deliberative. It's essential feature is the use of working memory in which information is kept in an active state because it is useful in ongoing processing (36). The employment of working memory is time-consuming, attentionally-demanding, and energetically expensive, so Type 2 thinking is better suited to solving complex social problems that do not require an immediate response. This distinction is what Daniel Kahneman refers to as “thinking, fast, and slow” (37).

Sadness, which is a crucial symptom of melancholia, is well-known to promote a Type 2 processing style (16, 38). Many other symptoms of melancholia can be non-randomly organized around the time-consuming, attentionally-demanding, energetically expensive nature of Type 2 thinking (21). For instance, one will be unable to effectively engage in Type 2 processing if one is continuously distracted by thoughts of food or sex, so the symptom of anhedonia may help one engage in Type 2 processing without interruption. Also, chronic activation of the HPA axis tends to direct energy to the brain, which can be used to support Type 2 processing.

Furthermore, many of the neurological changes that support Type 2 processing—working memory, distraction-resistance, the reallocation of energy to brain activity, an attentional focus on a threat or problem, and a loss of interest in other activities (anhedonia)—are coordinated by an increase in serotonin transmission to various forebrain regions. The idea that melancholia involves an increase in serotonin transmission may seem like the fatal flaw that refutes our hypothesis, because the conventional wisdom is that depression is associated with a reduction in serotonin transmission. However, the low serotonin hypothesis arose as a result of trying to explain how ADMs reduce symptoms, and it is widely recognized that their mechanisms of action are not well-understood (39). We have reviewed extensive evidence elsewhere that serotonin is upregulated in unmedicated depressed people, and in rodent models of depression (17).

In short, melancholia exhibits signs of adaptation for promoting Type 2 processing. We refer readers to our other work for a more in-depth treatment of this issue (16, 17, 21). Because Type 2 processing is analytical, we refer to this as the *analytical rumination hypothesis* (ARH) (16, 21). We have shown that the ARH applies directly to melancholia, but that it also may be useful in explaining atypical depression or other depressive phenotypes (21).

When an evolutionary biologist tries to tease apart the ancestral conditions that might have given rise to an adaptation, s/he engages in a process called “reverse engineering” in which the current manifestation is taken apart to see how the mechanisms works (an analogy is often made to deconstructing a watch to see what it was designed to do) (32). When attempting to reverse engineer a trait with unknown evolutionary origins, it is useful to follow the distribution of metabolic resources (energy), much as when “Deep Throat” advised Woodward and Bernstein to “follow the money” in Watergate. Evolutionary biologists “*follow the energy”* when they can.

There are at least three syndromes that involve depressed affect and a loss of interest in hedonic pursuits (anhedonia). When someone gets an infection, energy is directed away from cognition and the brain and toward the immune system. When someone is starving, energy is directed away from the immune system and growth and toward the maintenance of the vital organs, particularly the brain (17). When someone gets depressed (extrapolating from the classic melancholic form of depression) energy is directed away from the immune system and maintenance of vital organs and toward the cortex. These differential energy transfers are all coordinated by serotonin, a very ancient neurotransmitter that co-evolved with mitochondria (the energy-generating “blast furnaces” within each cell), and that is the target of nearly every ADM.

All the neurons in the brain that use serotonin as a neurotransmitter have their cell bodies in the raphe nucleus. The raphe nucleus is itself buried deep in the brainstem, suggesting that it developed a very long time ago in our ancestral past; serotonin is over 600 million years old and is present in almost all central nervous systems (40). When the raphe nucleus fires, it activates the amygdala, so as to keep the organism focused on the source of its current distress, as well as the hippocampus, so as to bring working memory online, the lateral prefrontal cortex, so as to make the organism resistant to distraction, the nucleus accumbens, so as to dampen down hedonic pursuits (anhedonia), and the hypothalamus, so as to tamp down growth and reproduction. In short, when the raphe nucleus fires it redistributes energy throughout the brain in a manner that facilitates rumination (17). *Avoidant learning is the effect that non-randomly organizes the features of depression and non-random organization must be a consequence of natural selection*.

Jeffrey Gray mapped out two coordinated neurobiological systems: (1) avoidance of threat, the behavioral inhibition system (BIS), largely noradrenergic in nature; and (2) the pursuit of pleasure (appetitive stimuli), the behavioral activation system (BAS), largely dopaminergic in nature (41). It is the latter that seems to be most directly suppressed in depression. Imminent threat requires an immediate response, whereas the pursuit of appetitive rewards can be delayed until the timing is propitious. Any organism must do two things as it goes through its day; it must get lunch without becoming something else's lunch, and the former always will take precedence over the latter (42). What is most relevant for our immediate discussion is that serotonin, the primary target of nearly all of the antidepressant medications, moderates the distribution of energy between inhibition (BIS) and activation (BAS) and as such largely coordinates the relative balance of these disparate types of activities and the affective syndromes they reflect.

## Question 3: What Is the Content of Rumination and What Is Its FUNCTION?

Clinicians tend to think of rumination as merely a symptom of depression or, even worse, a causal process in its own right (43). In point of fact, it is clinicians who have given rumination a bad name. Merriam-Webster's *New Collegiate Dictionary* defines rumination as to “go over in the mind repeatedly and often casually or slowly … to engage in contemplation” (44), whereas that same company's *Medical Dictionary* defines rumination as “…obsessive thinking about an idea, situation, or choice especially when it interferes with normal mental functioning; specifically: a focusing of one's attention on negative thoughts or feelings that when excessive or prolonged may lead to or exacerbate an episode of depression” (45).

Clinicians associate rumination with depression and assume that it serves no useful function, despite the fact that that is what the brain seems to be predisposed to do when loss or failure has occurred or is anticipated. Most episodes of depression remit on their own in the absence of treatment (something known as “spontaneous remission”) and that is not the case for most other non-psychotic disorders. Someone with a fear of heights tends to stay afraid of heights throughout his or her lifetime unless he or she takes specific action to resolve the fear (situational but not temporal) whereas someone who is depressed tends be depressed across situations until the episode remits (temporal but not situational) which it almost always does. That brings us to the question: Why do depressions go away? In our ancestral past, before the advent of treatments, something must have accounted for what appears to be such “spontaneous remission.”

Normal emotions are evolutionarily ancient, and they evolved because they motivate adaptive responses to specific situations. Positive emotions motivate the pursuit of fitness-enhancing opportunities (BAS), whereas aversive emotions motivate the avoidance of fitness-reducing harms (BIS). Normal emotions resolve when the opportunity or problem that triggered the emotion resolves (21). Because normal emotions promote adaptive responses to the situation that triggered them, they generate the source of their own resolution. The evidence that melancholia is an adaptation for promoting Type 2 thinking therefore suggests that depressive thinking may contribute to such “spontaneous remission” via resolving the triggering problem.

Depressive thinking (aka rumination) has self-blaming themes of worthlessness and culpability. How could it be adaptive to have such thoughts after a loss or failure? Is that not self-defeating? How could focusing on one's own inadequacies help one resolve the problems caused by loss or failure? Indeed, it might seem maladaptive to engage in any cognitive effort about a loss or failure, because one cannot reverse time to avoid an event that has already occurred.

Our starting point is that thinking about a loss or failure is not wasted effort if it helps you redress a social problem that still continues or to avoid similar such events in the future. Redressing such events or avoiding them in the future requires understanding why the loss or failure happened, which in turn requires reconstructing the causal chain of events that led to the bad outcome. Moreover, not all causes are equal. Those causes that you could have done nothing about are of less use than those for which you could have taken preventable action. Analyzing the chain of events that led to a loss or failure and focusing on those points in the causal chain where one could have taken preventable action, is called a root cause analysis (RCA). Such an RCA is often employed to reduce the risks of mistakes and errors in the business world and health care.

RCA requires Type 2 processing because reconstructing the causal chain of events that led to the failure or loss will occupy working memory and our capacity for storing things in working memory is no greater than that for our primate cousins (46). Additionally, the load on working memory is exacerbated by the fact that one must consider different hypothetical actions that could have been taken to understand if any one or more could have prevented the loss or failure.

One outcome of RCA is the development of *upward counterfactual thoughts* (21) that take the following form: “If only I had done X, then harmful event Y would not have happened to me.” They are *counterfactual* because they reflect a belief about how the present situation could have turned out differently if different action had been taken. And they are *upward* because they focus on how the situation could have turned out better than it did. Counterfactual thoughts reflect a belief about what caused the harmful event, and the action that could have been taken to prevent it. Clinicians will recognize that such thoughts are common in their depressed patients. Note also that counterfactual thoughts often have a self-blaming bias. When redressing an existing problem, it is often helpful to take responsibility for one's own actions and when attempting to avoid similar losses or failures in the future, a biased search for self-blaming causes is more adaptive than blaming external events because one has the most control over one's own future actions (47).

A natural explanation for the guilt and remorse that occur in melancholic rumination is that they display regret for past actions and motivate the search for root causes when preventable action could have been taken, and they lead to upward counterfactual thoughts that help one reduce the risk of recurrences. As we describe in detail elsewhere, the melancholic symptoms of low self-esteem (worthlessness) and pessimism (negative expectations) also play motivational roles in the search for root causes and the development of upward counterfactual thoughts (21). As Leary and Baumeister have described beliefs about one's character (“I am worthless” or “I am unlovable”) are beliefs about oneself that have a social component (48). Sociometric theory proposes that self-esteem evolved to monitor social acceptance, not so much as to maintain self-esteem, and that it serves to detect cues indicating that the individual is not adequately valued. As such, it motivates behaviors that enhance one's value to important others in one's social world. Beliefs about the self that lack any social context are unlikely to be acted on by evolution, since it is natural selection (operating through inclusive fitness) that shapes how organisms interact with their environment. Beliefs must affect social behavior in order to be shaped by evolution.

In summary, Type 2 avoidant learning of harmful events non-randomly organizes all the major symptoms of melancholia (sadness, anhedonia, chronic HPA activity, rumination, guilt, worthlessness, pessimism) and quite possibly serves a social function in interpersonal conflict. The promotion of Type 2 avoidant learning is therefore the evolved function of melancholia (21).

## Question 4: What Is the Relationship Between Rumination and Spontaneous Remission?

As just described, melancholic rumination often focuses on understanding the causes of problems, with a particular focus on self-blaming causes. This is hypothesized to be useful in figuring out how to solve those problems (e.g., redressing complex social problems that already exist and taking preventative action that reduces the risk of recurrences). Once those problems are solved, the depressive episode is predicted to resolve. In other words, under the ARH, depressive episodes are predicted to resolve through a sequential two-step rumination process (16, 49, 50). In this model, depressive symptoms first promote RCA, which then promotes problem-solving analysis (PSA). In turn, PSA leads to the resolution of the triggering problem or reduces the likelihood of its recurrence and thus reduces depressive symptoms, thereby contributing to spontaneous remission, which is, in fact, anything but “spontaneous” but instead the outcome of a process. In engineering terms, the ARH predicts that melancholia is part of a “closed system” that responds to disturbances and then returns the system to equilibrium. Problems drive depression that in turn drives causal analysis that then facilitates problem solution that then resolves the depression. In essence, spontaneous remission can be viewed as an “unaided resolution” in which depression does its job (motivating steps that lead to problem resolution) and then goes away, much like a fever resolves when it has contributed to the death of the invasive pathogen.

We have found consistent support for this model in a series of papers involving both clinical and non-clinical samples (51–53). Specifically, RCA is more temporally proximate to depression than PSA (52), and it acts as a mediating variable between depression and PSA (50, 52), both of which are consistent with our sequential model. We also found consistent evidence that PSA exerts negative feedback on depressive symptoms, which suggests that PSA may play a role in spontaneous remission (50, 52). Finally, in a sample hospitalized for major depression, we found that higher levels of PSA 1 week after admission were associated with lower levels of depressive symptomatology 5 weeks later, also consistent with spontaneous remission (53).

## Question 5: Why Do Depressed People Often Have Recurrences?

As noted earlier, depression appears to be far more common than our retrospective epidemiologic studies would lead us to believe. According to cohort studies that follow samples prospectively from birth, its actual incidence may be up to three times higher than standard psychiatric estimates, and the bulk of those additional instances occur in response to major life stressors among people who do not go on to become recurrent (6). These are the persons referred to by Monroe and colleagues as “depression possible” and that designation is virtually synonymous with “species typical.” Few individuals ever make it into treatment in their first episode unless it goes on long enough to be considered chronic (currently defined as 2 years or more). What this suggests is that the majority of individuals who ever get depressed get out of their episodes on their own with no subsequent recurrence. That suggests the operation of some kind of evolved adaptation that serves its function and then desists. That is the very definition of a “closed system” in engineering terms, and that is exactly how the ARH is presumed to operate.

What to make of the individuals who are “recurrence prone”? Multiple explanations are possible. First, according to Monroe and colleagues, there is no reason to suspect that simply experiencing an episode of depression increases an individual's risk for having another (the widely accepted “kindling” hypothesis is based solely on the observation that it is easier to identify a precipitant for initial episodes than for later ones). Rather, according to Monroe and colleagues, the fact that the number of prior episodes predicts the likelihood of subsequent episodes is simply an artifact of mixing “depression possible” and “recurrence prone” individuals in heterogeneous samples (6). It is likely that elevated risk either can be inherited or acquired (the latter likely prior to adolescence), but it does not necessarily grow across repeated episodes.

Second, if melancholic depression is a normal emotion, the quandary dissipates, because all emotions are recurrent experiences. As humans, we experience love, anger, fear, and most other emotions multiple times in our lives, and it is no mystery. Recurrences of emotions take place because people are exposed to the events that trigger them multiple times in their lives.

Third, people also appear to differ in their capacity for *experiential avoidance* (i.e., the use of distraction, thought suppression, self-medication, or other tactics to avoid experiencing painful feelings). Experiential avoidance is associated with worse outcomes from depression [for reviews, see: (16, 21, 54)], which suggests that a higher propensity for recurrences could be associated with a greater tendency to utilize experiential avoidance when one is depressed. In essence, if one does not learn from experience (painful though it may be), one is prone to repeating the same mistake.

Fourth, some problems may be so complex that their solution may require slowly grinding away at them over the course of years in bursts and bouts of intense melancholic mental activity, punctuated by periods of respite and rest. Why? Often, the only feedback people get that their mental model of their social world is inadequate is that they fail to achieve their social goals. However, it may not be obvious what aspects of their mental model are problematic. Does their whole understanding need to be revised, or does the model simply need to be tweaked? Usually, it is better to tweak the mental model unless substantial evidence indicates wholesale revision is required. After all, the current model is the product of years of experience and may have worked well in the past. The individual may need to develop different hypotheses about which parts of their mental model need to be refined, and then test them systematically until feedback improves. People who are prone to depression are not unique in terms of being conservative when it comes to changing their beliefs, that is a characteristic common to the species. New information that contradicts an existing belief is viewed with greater skepticism than information that confirms what one already believes (55). If “insanity” is doing the same thing over and over again and expecting different results, then the bulk of the human race is functionally “insane,” since most of us operate in that fashion. It is not that new ideas do not win out in the end (if they do a better job of representing the external realities), it is just that a critical mass of anomalies must accumulate and be noticed before an existing paradigm begins to shift. Having an alternative that can better account for the anomalies is usually required to facilitate such a paradigm shift (56).

Most depressed patients seen in clinical settings appear to have latent schema regarding unlovability or incompetence that get triggered by negative life events. The difference between the “depression possible” and the “recurrence prone” may be the ease with which subsequent episodes get triggered (patients with Axis II personality disorders appear to be at particular risk since they tend to engage in “compensatory strategies” to protect themselves from loss or failure that annoy other people), but any and all would benefit from the type of careful Type 2 thinking (rumination) that moves the process along to resolution. People who have lost a loved one through no fault of their own (grief) still have many realistic problems to resolve and those who come into adolescence with a latent belief that they are unlovable are especially prone to making errors in relationships or interpreting occasional conflicts that arise as reflections of their worth.

Similarly, people who have experienced a major vocational setback or achievement-related failure would be well-advised to consider what steps if any they could have taken to avoid that failure as a prelude to what steps they will take in the future to move their prospects along and those who are schematic for incompetence even more so. Physicians who make medical errors often respond by becoming depressed and as a consequence exercise greater care in their future practice (21). Once again, this process is helped along by an apparent shift into Type 2 thinking (rumination) that is motivated by their affective distress.

In keeping with the notion that depression is an adaptation that evolved because it served a function, it is interesting to note that the symptoms expressed tend to differ as a function of the triggering life event; death of a loved one and romantic breakups elicit sadness, anhedonia, appetite loss, and guilt (with the latter restricted to breakups), whereas chronic stress and failure are associated with fatigue and hypersomnia (57).

The majority of people that we see in treatment (and by extension in clinical trials) are “recurrence prone” who themselves represent a minority of the people who ever get depressed. A major feature of many such patients is the operation of latent schemata that lie dormant until activated by negative life events (20). The beliefs at the core of those schemas are often “stable” trait theories about the self (“unlovable” for those concerned with affiliation and “incompetence” for those invested in achievement). We put “stable” in quotes because we think these are actually conditional beliefs. Patients would not bother to come to treatment if they did not think these propensities could not be changed or at least worked around. From an evolutionary perspective, it is adaptive for people who get depressed to consider ways in which they may have contributed to the problems that they face. That is part of the root cause analysis, and if their actions contributed in any way to the genesis of the problems, then those actions can be avoided in the future.

One of the major strategies in CBT is to encourage the patient to consider other explanations than a trait-like defect in the self (conditional or otherwise), and most often that is that they were simply pursuing the wrong strategy. This is what Salkovskis refers to as pitting “Theory A” (“I am defective”) vs. “Theory B” (“I chose the wrong strategy”) (58). As we indicated above any consideration of the causes of a problem should include consideration of the role one might have played since it is one's own behavior that is easiest to modify in future problem situations. Distress drives the search for causes; changing behaviors is often the solution. As indicated above, self-referential beliefs are best understood as reflecting one's perceived value to others and the behaviors they motivate are those that impact on one's social environment (48).

We know that people who get depressed tend to generate more life stress in terms of events that could be “dependent” on their own problematic behavior (e.g., a divorce or getting fired as opposed to the death of a loved one), a phenomenon referred to as stress generation (59). Since these studies are based on clinical samples and since clinical samples tend to skew toward the “recurrence prone” what we think that means is that people who have an underlying diathesis (inherited or acquired) tend to generate behaviors that increase the number of stressors that they face. It is not that they necessarily confront more stressors because they tend to get depressed (although that likely happens too since people cope less well when depressed) but rather that they get depressed more often than other people because they inadvertently generate more life stress.

There is nothing about stress generation that is incompatible with an adaptationist perspective. If some people inadvertently generate life stressors, they would be expected to get depressed more often than others who do not, and that is exactly what appears to happen. We also know that individuals who are prone to making internal, stable, global attributions for the problems that they encounter are more likely to become depressed when things go wrong than those who tend to make other types of causal attributions (60). What we think this means is that individuals with an underlying diathesis (inherited or acquired) are at greater risk of becoming depressed in response to life stressors that would not be depressogenic for others who do not share that underlying diathesis (the “depression possible”). Again, there is nothing about the notion that having a particular attributional style is depressogenic that is incompatible with an adaptationist perspective or evolutionary theory. If having a particular attributional style increases the likelihood of becoming depressed in response to the same negative life event then that simply means that those individuals will have more need to shift into Type 2 thinking in order to solve what they perceive to be a bigger problem than other people perceive that problem to be.

What we think this all means in aggregate is that depression is an evolved adaptation that works both for those people who confront only the occasional major life stressor (the “depression possible”) and for those people who inadvertently generate an overabundance of negative life stressors or who overreact to less severe stressors (the “recurrence prone”). It is just that it will have to “kick in” more often for the latter. There is no evidence that episodes last any longer (on average) for one group than the other or that spontaneous remission is any more or less likely to occur for either. What is likely is that people who are “recurrence prone” are more likely to find their way to treatment since they know from prior experience that even though each episode tends to go away on its own it often takes many months to do so. For the “depression possible” clinical intervention may not be necessary but (depending on its nature) not necessarily problematic. CBT may be overkill (analytical rumination will likely help them resolve the triggering problem before it occurs to them to enter treatment) whereas ADM may be unnecessarily iatrogenic (if it prolongs the episode and leads to relapse when the medications are taken away) (22).

For those among the “recurrence prone” CBT is likely to be preferred for those who will respond to it (not all will) since it seems to facilitate the processes that depression evolved to serve with respect to resolving the problem that triggered the episode in the first place and to have an enduring effect that reduces risk for future episodes (22). We think this is a consequence of either dismantling existing depressogenic schema (accommodation) or teaching compensatory skills (compensation) that allow patients to short-circuit the episode before it starts (61). For those among the “recurrence prone” who do not respond to CBT or some other empirically supported psychosocial interventions like behavioral activation (BA) or interpersonal psychotherapy (IPT), then ADM may still be the treatment-of-choice by necessity. In an earlier trial we found that patients with depressions superimposed on Axis 2 personality disorders were more likely to respond to ADM than to CBT but especially likely to relapse when ADM was discontinued (62). For such patients, who are particularly likely to engage in behaviors that generate problems in affiliative and achievement related endeavors, short-term psychotherapy might not be sufficient. There are things that can be done with such patients in lieu of or in addition to medications, but they generally take months or years instead of weeks and require addressing the behavioral strategies that patients have developed to compensate for perceived inadequacies (63).

There are several types of life events that appear to increase risk that someone will develop a depressogenic schema, childhood trauma, and death of a parent among them, and the greatest lasting impact seems to occur when those events occur prior to or early in adolescence. The strategies used in CBT largely revolve around encouraging clients to use their behaviors to test the accuracy of their beliefs (run experiments) and catch themselves when they start to slide into Type 1 thinking, such as “all-or-none thinking” or the rigid application of “shoulds” (64). None of this would work if the patient were not capable of generating alternative explanations for a given negative event (“Theory A vs. Theory B”) and weighing the evidence for and against each.

We argue elsewhere that sadness motivates introspection, and that such introspection is a useful tool when things go wrong, especially when that negative event could have been the consequence of one's own problematic behavior (21). Negative affects clearly play an important motivational role. If you did not feel distress in response to something going wrong, you would not be motivated to fix whatever caused the problem. To the extent that the stress generation hypothesis is true, then those who are “recurrence prone” likely carry in their heads an internal recipe for making inadvertent mistakes in life, likely as a consequence of generating self-fulfilling prophecies in which their own negative beliefs lead them to engage in self-defeating behaviors that generate the very outcomes that they fear (20).

## Question 6: Does Cbt Disrupt Rumination or Make It More Efficient?

If depression is an evolved adaptation that serves to motivate efficacious problem-solving, then it is likely better to promote that process than to disrupt it. From an adaptationist perspective *it is not the distress that is the problem, but rather the problem that generated the distress*, and it is the problem that needs to be resolved. If so, then thinking about the triggering circumstances in a careful and deliberative fashion (rumination) is one step in the process of problem resolution. A case can be made that CBT teaches people how to ruminate more efficiently (64). Everyone engages in both Type 1 (rapid judgments dominated by heuristics and biases) and Type 2 (careful, methodical, analytical deliberation) thinking, it is just that depression tends to motivate more of the latter. Much of what passes for positive self-esteem in those who are not depressed is based on positive illusions and such an “illusory glow” only works when things are going well (65).

What we think we do in CBT is to take advantage of the depressed patients' proclivity for ruminating about the problems in their lives. However, ruminating about the causes of one's problems does not necessarily mean that the causes considered will be correct or that the solutions generated will necessarily be efficacious. Although most episodes will resolve over time (often as a consequence of the one's own efforts at resolution whether recognized or not), some patients get “stuck” and when they do it is usually because they have settled on a causal explanation that focuses on some defect in the self (incompetence or unlovability) that does not readily suggest a behavioral solution that will solve the problem.

This is wholly consistent with our evolutionary view that suggests that negative characterological explanations are a normal part of our evolved psychology in response to serious failures and losses. For instance, as we describe in more detail below (see Question 9), characterological explanations may have a motivational function (21). Moreover, there are a number of normal factors and constraints on human cognition that may make it difficult for people to see non-characterological explanations following losses and failures (20, 21) and may make them appear to be cognitively “stuck.”

In this context, CBT may be particularly useful in helping people identify and consider non-characterological alternatives. If the essence of an adaptationist theory is that depression is an evolved adaptation that motivates the person to ruminate about the causes of their distress so that an efficacious solution can be found, the essence of cognitive therapy is helping persons who get stuck along the way by helping them correct errors in their thinking; that is, to ruminate more efficiently and to a better end.

One of the authors (JAT) worked with a 30-something graduate student in the natural sciences who has been chronically depressed for 3 years. The problem was that he was in a very difficult program of studies that few of us could master, not that he was depressed as a consequence. The patient had come to believe that he was a “failure,” and his psychotherapist was pushing the author (a psychiatrist) to medicate the patient so as to resolve the depression. There was nothing “characterological” about his depression and his proclivity to look for self-referential explanations likely served a motivational purpose. That said, it was likely that the cause of this instance was that his program was simply very difficult and something few of us could master.

Integral to that process is encouraging patients to ask themselves three questions whenever they catch themselves having an automatic negative thought: (1) ***evidence:***what is my evidence for that belief? (2) ***alternatives:***are there any other explanations for that event other than the first one I came up with? and (3) ***implications:***are the real implications as dire as I first presumed? In effect, CBT therapists do not so much try to disrupt rumination as to facilitate it and to give it structure (64). The alternatives question, in particular, invites the patient to consider multiple possible explanations for the problems that they face. This is analogous to what Salkovskis has described as pitting Theory A (the patient's explanation for his or her distress that in the case of depressed patients usually focuses on some kind of perceived defect in the self) vs. Theory B (an alternative rationale that typically looks to see if the patient has simply adopted the wrong behavioral strategy) (58). The evidence question then prompts the patient to review the existing facts and encourages him or her to gather new information to test between the competing theories, often by virtue of conducting behavioral experiments in real life that the therapist cannot control. Finally, the implications question prepares the patient to parse out what the likely consequences (if any) will be of the problems that they face. The goal is not simply to relieve distress (that can be done more rapidly with ADMs), but rather to first accurately identify the cause of the problem that is causing the distress (root cause analysis) and then come up with a plan to resolve it (problem solving analysis). This process is wholly consistent with the ARH. Moreover, it teaches the patient a strategy that they can follow in future instances if they do again become depressed, and that is likely what accounts for CBT's long-term enduring effects (66).

In an earlier article we described two patients who both were treated in this fashion (20). Both were severely depressed at the time they started treatment, but one was a patient with a relatively uncomplicated case of depression whereas the other had a depression superimposed on a host of problematic interpersonal behaviors that looked at first to be consistent with a borderline personality but turned out to be more a case of complex PTSD. That being said, the treatment of each followed a very similar format (albeit requiring only a matter of months with the first and a matter of years with the second), with more purely behavioral self-monitoring giving way to training more efficient rumination (as described in the preceding paragraph) that was framed in each case around conducting a set of ongoing tests of opposing theories (Theory A vs. Theory B).

The first patient was a 40-something sculptor who had lost his job teaching in a liberal arts college about 3 years earlier through no fault of his own when his entire art department was let go during an economic downturn. He had been working as a handyman in a condominium complex for the last 3 years following his dismissal and been depressed for the bulk of time, a fact he attributed to his “dead end” job. He viewed his distress as being a reality-based depression and could not imagine getting better until he was employed again in academia. Simply asking him to monitor his moods and activities between the first and second session quickly revealed that he felt his best when he was at work and his worst when he was at home on the weekends and in the evenings thinking about how much he hated his “dead end” job. It also quickly became apparent that he blamed himself for being stuck in his current situation, which he attributed to being an “incompetent loser” who “always screwed things up” (Theory A), the evidence for which being that he had yet to apply for another teaching job or pay his taxes during the last 3 years while depressed. Simple behavioral strategies such as graded task assignment (breaking a big task into component steps and focusing on completing one step at a time) were used to first help him get to a traveling art exhibit with his wife on the weekend and then put his portfolio together and start to apply for jobs. These simple behavioral experiments were used to test between his belief that he was an “incompetent loser” (Theory A) vs. the notion that he was simply choosing the wrong “behavioral strategy” (Theory B) and getting overwhelmed by the task. This culminated in an incident in which he was able to catch his own automatic negative thoughts and correct them on the fly by using both the alternatives and evidence questions when he found himself stymied by the magnitude of the task involved in organizing his financial records so that he could pay the back taxes that he had ignored for the last 3 years [“I have gotten filing and other stuff done in recent weeks (evidence) when I take a big task and break it down into smaller steps that I can do one at a time (alternatives)”]. Finally, he used the “implications” question to reason that the IRS would be unlikely to send him to jail if he came in voluntarily, something he confirmed with two anonymous phone calls from two separate phone booths in two different twin Minnesota cities.

The second patient was far more complicated, and her issues took far longer to resolve, but therapy progressed through a similar process across a far more extended time frame. She “conned” her way into treatment having gone to *ClinicalTrials.gov* to discover that the particular study in question screened out patients who met criteria for borderline personality disorder and then borrowed a DSM from a graduate student friend to see what she had to deny at intake in order to make it into the study. She was screened in and randomized to CBT and assigned to one of the authors (SDH) for treatment. At her first session she announced that she had been deeply damaged by something that happened to her as a teenager (and that she did not want to talk about in therapy) and as a result had become a “bad” person who invariably tore apart any romantic partner with whom she got involved. She further made it clear that she had no intention of following the study protocol that called for a maximum of 24 sessions over 16 weeks (with an emphasis on teaching her how to do the therapy for herself so as to make the therapist obsolete); what she wanted instead was for someone to see her four or five times a week for the rest of her life in order to keep her “predatory” relational tendencies in check. She further stated (in response to her therapist's quizzical look) that that should not be that great a burden since she was twenty-nine and did not plan to live past her thirtieth birthday in 6 months. She closed by stating that she was an incorrigible liar, and that the therapist could not believe anything she said, asking if that would be a problem for the therapy. She was somewhat mollified (and bemused) when her therapist told her that it would not be a problem, since his job was not to solve her problems but rather to teach her skills that she could apply to do so if she chose, and that he could teach her those skills as readily working with any fabrications that she made up as with the actual truth. Whether she was honest or not was irrelevant, since there would be by necessity coherence in the stories that she told (her thoughts, feelings, physical reactions, and behavioral impulses would invariably be shaped by evolutionary pressures to form an integrated “whole body” response) and that was all that was needed for the therapist to teach and the patient to learn to apply the skills.

**Figure 1:**
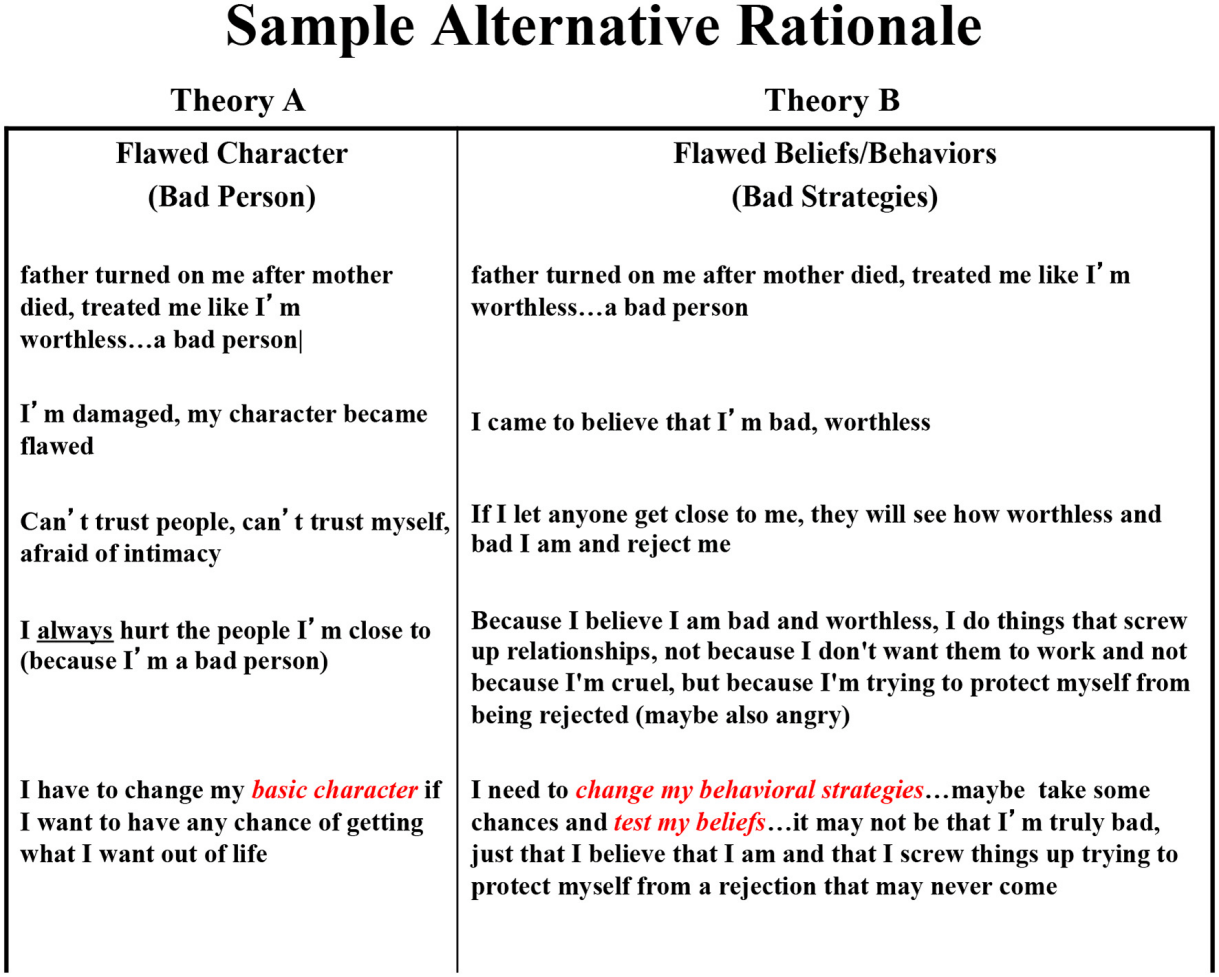
Sample alternative rationale.

Over the first few sessions the patient and therapist sketched out competing theories regarding what had happened to her and how it had affected her life. Theory A (the one she brought into therapy) was that her father's lack of concern about what had happened to her as a teen taught her that she was worthless and someone that no one would ever love. She responded to this depressogenic schema by adopting a set of compensatory strategies, dissimulating and manipulating her partners in relationships in a desperate effort to secure some small measure of affection before the inevitable rejection, which she forestalled by beating her partner to the punch; she had just run off from her husband of 2 years to join a man she had met over the internet for an affair that lasted less than a week. After some probing of those and other previous relationships (all of which came to a bad end) the therapist developed an alternative Theory B that again started with her father's indifference to her teenage trauma but then proceeded through the notion that she only came to believe that she was unlovable and as a consequence had adopted a series of strategies (manipulate and dissimulate) that she used to compensate for her perceived defects, and it was these strategies, and not any presumed “unlovability” that led to the demise (at her hands) of any relationship she entered. A single-paged two-column depiction of “Theory A vs. Theory B” became the basis for the rest of the therapy, and every behavioral experiment that she ran was cast as a test between the competing formulations (see Figure 1). Was the problem that she was defective (a stable trait that would be difficult to change) or that she adopted a set of problematic behavioral strategies in an effort to compensate for her beliefs (that would be easier to change)?

As might have been expected, the traumatic event was a gang-rape by her father's drinking “buddies” in her own home less than a year after she lost her mother to cancer. Her father's total lack of concern when she told him about the rape led her to run away from home and set her off on a several year binge of romantic misadventures (first eloping with her high school boyfriend that his parents quickly got annulled) and then a series of failed relationships that culminated in her deserting her husband and ending up back at the inner-city school where she had done her student teaching. Therapy proceeded through the standard CBT strategies (training in self-monitoring, behavioral activation, and cognitive restructuring) much as it had with the sculptor, but with a few additional twists. The client started making “anonymous” phone calls to the therapist in the middle of the night “just to hear his voice,” so he negotiated a deal in which he installed a phone answering machine in his office at work that she could call any time of the day or night on the proviso that he would not check the messages (if she had something that she wanted to discuss she could do it in their nearly daily sessions). She was very suspicious about what other people (including her therapist) were thinking about her (she thought that they thought that she was wild and promiscuous and out sleeping around every night) and often got angry and verbally hostile about those presumed “flights-of-fancy” on the part of the therapist, so he negotiated yet another deal in which he agreed to write down exactly what he was thinking when she became suspicious and show it to her if she chose, which she always did. It was often enough the case that what the therapist was thinking about was innocuous or embarrassing (to him) (“how long is she going to prattle on?” “what should I pick up for dinner on the way home”) that she came to trust the honesty of his report and question the accuracy of her own suspicions.

Therapy proceeded for several hundred sessions over the next several years with the frequency decreasing over time from virtually daily sessions to one or two a week and then spacing out to monthly then yearly visits. It took 3 months to persuade the patient to relive the traumatic rape with her therapist, but when she did it became clear that she took two meanings away from the event (and her father's subsequent indifference): first, that she would be of little value (and hence “unlovable”) to anyone in whom she had a romantic interest if she revealed what had happened to her (she had lost value as a potential partner because she had been defiled), and, second, that she found it so frightening to think that something so awful could happen to someone who had done nothing to deserve it (the “just world” hypothesis), that she found it more comforting to think of herself as a “bad” person who did the worst to others before they could do the worst to her. It took a series of carefully calibrated disclosures (first to female friend and then to her current and subsequent partners) wrapped around an anonymous survey of “eligible” young male soccer coaches her therapist sampled on her behalf, before she came to realize that others did not share her view of herself as irrevocably damaged as a consequence of the rape. What her romantic partners did take umbrage at was the way that she treated them, the dissimulation and manipulation that she used as compensatory strategies in an effort to preserve her relationships.

She had a particularly difficult time asking for what she wanted from her romantic partners, expecting them to “read her mind” and then getting angry with them and acting out when they did not meet her expectations. Considerable time in therapy was devoted to role playing making such requests in a triadic fashion in which passive non-initiation reflected a respect for her partner's wishes but not her own, aggressive hostility reflected a concern for her own wishes at the expense of her partner's, and finally an assertive request that respected both her wishes and those of her partner and opened the door for compromise in those instances in which their wishes did not coincide. Several years after therapy ended the patient agreed to videotape (shooting over the back of her head to protect her anonymity) her recollections regarding what she did and did not like about therapy for a behavior therapy conference. What she indicated (among other things) was that the aspect of therapy that she disliked the most were the repeated role plays (she found them annoying and anxiety provoking) but that that was the aspect of therapy that she found most helpful (since they let her practice approaching partners as equals).

The question now becomes whether CBT actually works through the process of making rumination more efficient and honing behavioral skills that can be used to help patients get “unstuck.” It is easier to detect an effect than it is to explain it. That is because any effort to test for mechanisms of action necessarily involves a three (or more) variable causal chain, and any experimental design can only test the causal impact of the manipulated variable (for example treatment) on either the mechanism or the outcome but not both simultaneously (67). We can and do test for mediation in our designs, but such efforts are by necessity correlational in nature. The link between purported mechanism and the outcome of interest can only be established with any real confidence if we can institute multiple independent manipulations of the mechanism itself [see for example the elegant program of research instituted by Maier and colleagues to specify that it was a descending pathway from the prefrontal cortex to the raphe nucleus that determined whether rats exposed to escapable stress behaved in a helpless or resilient fashion (68)]. By way of analogy, efforts to test for mediation in the context of randomized controlled trials (RCTs) of different treatments are similar to the shadows cast on the wall of Plato's cave; at best they reflect the movements of the people sitting around the fire, but they are not the individuals themselves. Moving the shadows on the wall may not have a causal effect but moving the people will.

There are two steps in the causal chain from intervention to outcome that have been tested with respect to CBT (most of the work to be described has been done with cognitive therapy a particular type of CBT). The first are those components of the treatment manipulation that have a causal impact and are, in effect, its active ingredients (often referred to as treatment process). The second are the phenomena within the patient that are affected by those active ingredients for which we reserve the term “mechanisms.” Both play a causal role but are sequential in their temporal order such that the active ingredients (treatment processes) produce change in the patient mechanisms. Thus, in any efficacious treatment there is at minimum a four-variable sequence; some treatment manipulation (preferably with a randomized comparator) mobilizes active ingredients (processes) that in turn engage phenomena within the patient (mechanisms) that effect changes in the concerns that brought the patient into treatment (outcomes).

It has been shown that CBT works at least as well as ADM and better than pill-placebo (69) and that it has an enduring effect that reduces risk for subsequent symptom return following treatment termination (66). The same appears to be true with respect to acute response for BA (70) and IPT (71), although only BA as of yet has demonstrated an enduring effect that lasts beyond the end of treatment (72). It also appears to be the case that non-specific factors account for the “lions-share” of variance in change among patients with less severe depressions (73); specific effects emerge only among patients who are more severe with respect to both ADM (74) and psychotherapy (75). With respect to CBT, DeRubeis and colleagues have shown that adherence to cognitive and behavioral strategies in early sessions leads to change in symptoms that in turn leads to enhanced quality of the working relationship (76, 77). In effect, the best way to generate a good working relationship in CBT is to bring about rapid symptom change and the best way to do that is to adhere to its specific cognitive and behavioral strategies. With respect to underlying mechanisms, DeRubeis and colleagues found that change in cognition led to change in depression in CBT whereas change in depression led to change in cognition in ADM (78). Tang and colleagues have shown that patients who “get” the cognitive model are more likely to show “sudden gains” in treatment (rapid drops in symptoms) and also are less likely to relapse than patients that show comparable gains in a more gradual fashion (79) and Strunk and colleagues have shown that those patients who best internalize the compensatory cognitive and behavioral skills taught in CBT are those least likely to relapse following termination (80).

This is all consistent with the notion that CBT works “from the top down” with higher cortical processes overriding more emotional processes that emanate from lower limbic regions whereas ADM works “from the bottom up” in the opposite fashion (81). This also is consistent with work by Mayberg and colleagues who found specificity of change in cortical vs. limbic regions following CBT vs. ADM (82). That is wholly consistent with an adaptationist perspective given that energy is deployed to the cortex to facilitate slow and deliberate Type 2 ruminative thinking. CBT requires that patients engage in a careful logical reconsideration of their beliefs and the problems that they face; that is clearly something that they could not do if their “brains were broken.” Much in CBT is compatible with an adaptationist evolutionary perspective.

## Question 7: Stigmatize Vs. Validate?

It is never good to stigmatize the patient and that is one risk that CBT can run. There is an ironic exchange between Aaron Beck and the woman who was roleplaying the patient in the classic Mia videotape. After she described concerns that her son was stealing things in school and that her husband may be having an affair, both of which she attributed to her failure (as a mother and as a wife), Beck started to describe the cognitive model (that her thoughts might be in error) and she slapped her head and said, “So even my thoughts are no good!” As we describe in greater detail in our treatise on “Disordered Doctors or Rational Rats” it is likely preferable to describe depression as a normal (if unpleasant) evolved adaptation in a manner that validates the patient's emotional experience (21). It is possible to differentiate between beliefs that may not serve the patient well and the emotions that they generate, and it is axiomatic to say something along the line of “if you think you are to blame for your son's stealing or your husband's (suspected) infidelities, how could you not feel sad?” Where an adaptationist perspective would separate from a conventional CBT response is in suggesting that even negative self-referential beliefs may play a useful evolutionary role in exploring the possible causes for the problems that the patients face whether they turn out to be accurate or not. One of the authors (JAT) will often say to patients, “Given what you just described, we would be far more worried about you if you were not depressed. We'd be waiting for the other shoe to drop, for you to sink into substance abuse or worse. Your depression did its job. It stopped business as usual amidst this complex calamity and focuses your attention on it.” It is “only human” to consider all the possibilities when things go wrong and, from an adaptationist perspective, the opening gambit that motivates a search for a solution.

The essence of root cause analysis is to explore all possible causes whether flattering or not (21). That is neither an instance of disease nor disorder but a step along the process of understanding the causes of the problem as a prelude for coming up with a solution. In CBT, the therapist is schooled against invalidating the patient's affect experience but quick to look for inaccuracies in his or her beliefs. An adaptationist perspective would suggest that recognizing the value of considering all possible explanations, including those not flattering to the self, is what the human brain is designed to do and not an indication of dysfunction. Once that is done patients can proceed to generate a range of alternative explanations and gather evidence to test among them but do so in the knowledge that there is nothing inherently wrong with their brains (20).

## Question 8: Is It Better to Treat Depression With Adm or Cbt?

ADM and CBT clearly work and have comparable short-term efficacy (on average). About 30% of patients with more severe depression are more likely to respond to ADM than to CBT and a different 30% of the more severely depressed show the opposite pattern (83). Among patients with less severe depressions there is little evidence of specificity; neither ADM nor CBT separate from non-specific controls like pill-placebo or supportive psychotherapy (74, 75).

What CBT has that ADM does not is an enduring effect that protects against relapse (the return of symptoms associated with the treated episode) following treatment termination (66) and possibly recurrence (the onset of wholly new episodes) relative to patients kept on ADM to the point of recovery (72, 84). Current convention in psychiatry is to keep patients with a history of chronic or recurrent depression (about 85% of all patients) on ADMs indefinitely. There is no indication that ADMs do anything to reduce future risk once you stop taking them and reason to think they might have an iatrogenic effect that prolongs the life of the underlying episode (22).

Modern psychiatry sees depression as being caused by a malfunction in the brain and this is the basis for the widespread use of ADMs (and especially the SSRIs) (39). However, ADMs are evolutionarily novel drugs and an adaptationist perspective would predict that they cause Wakefieldian disorders by interfering with emotional or physiological adaptations. At the least they should undercut the motivation to resolve the problems that triggered the episode in the first place in a manner akin to anesthetizing the squid so as to minimize the pain of surgery (31). If depression evolved to motivate the search for a resolution to the problem(s) that generated the distress, then simply medicating the distress may undercut that motivation. (One of the authors once had a patient that he had put on medications tell him, “I am no longer depressed but I am still married to the same abusive alcoholic.”) The cognitive and behavioral therapies, on the other hand [including both cognitive therapy and problem-solving therapy (PST) and related third-wave behavioral interventions like acceptance and commitment therapy (ACT) and behavioral activation (BA)], are all focused on problem resolution rather than simply dulling the distress.

All antidepressant medications (ADMs) produce an initial increment in the amount of neurotransmitter in the synapse. The older monoamine oxidase inhibitors (MAOIs) do so by inhibiting degradation of all three relevant neurotransmitters (serotonin, norepinephrine, and dopamine) in the presynaptic neuron, whereas the tricyclic antidepressants (TCAs), selective serotonin reuptake inhibitors (SSRIs), and serotonin-norepinephrine reuptake inhibitors (SNRIs) do so by blocking reuptake into the presynaptic neuron (85). Because all the different types of ADMs are believed to share a common downstream mechanism (an increment in the amount of neurotransmitter in the synapse that increases the likelihood that an impulse will be propagated along the post-synaptic neuron whatever that may be), it was long believed that depression was a consequence of a deficit in extracellular neurotransmitter that ADMs corrected (86).

In point of fact, no such deficit exists. It is exceedingly hard to measure neurotransmitter levels in the synapse of a living human being, so Barton and colleagues inserted a catheter in the jugular vein to assess serotonin turnover by measuring its principal metabolite exiting the brain. Contrary to expectations, unmedicated depressed patients showed elevated levels of serotonin turnover relative to normal controls, whereas patients stabilized on therapeutic medication doses had returned to the normal range (87). This presented something of a paradox with regard to the widespread belief that ADMs work by correcting a deficit in neurotransmitters in the synapse; if neurotransmitter levels are already elevated in the synapse, how is it that increasing them further (up to four times the levels found in nature) can reverse an existing episode of depression?

The resolution of this paradox requires a more sophisticated understanding of the way that medications work and their interplay with the underlying homeostatic regulatory mechanisms. In a classic monograph published just before the turn of the last century, Hyman and Nestler noted that most medications (ADMs included) do not produce their effects directly, but rather by triggering pushback from the mechanisms that maintain internal homeostasis (88). This principle holds not only for medications with psychiatric properties, but also for drugs of abuse; external administration of opiates suppresses internal synthesis of endorphins leading first to tolerance and then withdrawal when that external supply is cut off. As Andrews and colleagues note, when a patient starts on an ADM, the initial effect is to increment the amount of neurotransmitter in the synapse for the first week or two (during which time there often is an exacerbation of symptoms) before the internal homeostatic regulatory mechanisms kick in and suppress synthesis in the presynaptic neuron and post-synaptic sensitivity (17). As a consequence, neurotransmitter levels are reduced, and the medicated patient returns to “normal” levels as Barton et al. observed (87).

We now return to just what it means to have elevated levels of serotonin in the synapse. Norepinephrine and dopamine are the other two biogenic amines that serve as neurotransmitters that are involved in depression and both are largely modulated by serotonin. Norepinephrine is largely involved in the regulation of the stress response that underlies the BIS, whereas dopamine is largely involved in the pursuit of appetitive rewards that underlies the BAS. As previously described, all the neurons in the brain that use serotonin as a neurotransmitter have their cell bodies in the raphe nucleus (deep in the brain stem) and when they fire, they play a major role in the distribution of metabolic resources across the brain (and the body too). When the raphe nucleus fires, the brain is primed for immune response (if infected), maintenance of vital organs (if starving), or rumination (if melancholic). ADMs provide symptomatic relief, but they do not necessarily resolve the problem that triggered the depression in the first place. In effect, ADMs may anesthetize distress at the expense of leaving the individual more vulnerable to predation.

Moreover, when ADMs suppress symptoms, they may do so at the cost of locking down the very underlying homeostatic regulatory mechanisms that otherwise would have shifted back toward normal baseline levels once the problems were resolved (the process referred to as “spontaneous remission” but that is likely anything but spontaneous). One of the symptoms that ADMs suppress is rumination, and if (as the ARH suggests) its component steps (causal analysis facilitating problem solving) might otherwise have led to a solution, that “natural” healing process will not occur. Moreover, to the extent that ADMs “work” by perturbing the homeostatic mechanisms that regulate neurotransmitters, there is likely to be “push back” when taken away.

The process of pushing back on those homeostatic regulatory mechanisms has been termed “oppositional perturbation” by Andrews and colleagues and likened by analogy to compressing a coiled spring (89). This leads to the prediction that the more a medication class perturbs the underlying homeostatic regulatory mechanisms, the greater the risk of relapse when the medications are taken away and that is exactly what happens. Risk for relapse following remission on pill-placebo (no direct effect on neurotransmitters) is only about 20%, but doubles to 40% following remission on SSRIs that are serotoninergic only. That risk rises to over 50% for SNRIs and TCAs that also affect norepinephrine and to 75% for MAOIs that also affect dopamine (89). This is impressive accuracy for a prediction derived from adaptationist evolutionary theory.

This leads us to suspect that ADMs may suppress symptoms (a purely palliative effect) at the expense of prolonging the length of the underlying episode (20, 22). It is common practice in psychiatry to keep patients on ADMs for 6–9 months following initial remission to forestall relapse (the return of symptoms of the treated episode) on the presumption that the underlying episode will run its course (that spontaneous remission will proceed). However, if the notion of oppositional perturbation is correct, then the underlying neurobiology will be locked in place and patients will stay at elevated risk for relapse (about 3–5 times greater than the risk of recurrence – the onset of a wholly new episode) for so long as they stay on medications (90). In point of fact, the vast majority of patients are kept on ADMs for over 2 years and at least a quarter for over a decade (91) and current psychiatric guidelines call for keeping patients with a history of chronic or recurrent depression (the vast majority of clinical patients) on ADMs indefinitely (92).

Finally, the long-term consequences of ADM use actually may be deadly (93). The serotonin transporter is expressed in many peripheral organs and tissues (17). Serotonin evolved in the ancestral mitochondrion – the energy powerhouse in cells – and it has mitochondrial functions. What that means is that serotonin may have important effects on the metabolism of peripheral cells outside of the brain and that blockade of the serotonin transporter could cause Wakefieldian dysfunction in those cells. The diffuse set of side effects caused by SSRIs (gastrointestinal, cardiovascular, platelet function, sexual, and developmental) is consistent with that notion. Although SSRIs are presumed to be relative safe over the short run (one of the reasons they are preferred over the TCAs and MAOIs), a meta-analysis by Maslej and colleagues found a 30% increment in “all cause” mortality among patients free from cardiac illness (ADMs are mildly protective for the latter) (94). These findings need to be interpreted with caution since they were based on naturalistic studies that could have been confounded by other factors, but the effect was even stronger when initial levels of depression were controlled. Few industry-funded trials are continued for more than 6–8 weeks and multi-year maintenance trials are uninformative with respect to long-term risks since all patients start on ADMs. We know less about the long-term risks of ADMs than we ought to know, but what we do know is cause for some concern.

## Question 9: Why Do Depressed People Often Have Inaccurate Beliefs?

The ARH posits that depression is an adaptation that evolved to facilitate solving complex (often social) problems by virtue of motivating a switch from quick heuristic-driven Type 1 thinking into a more energy-expensive but carefully deliberative Type 2 thinking (rumination) (16). Cognitive theory suggests that depression is in large part a consequence of inaccurate beliefs and maladaptive information processing and that rumination is, at best, a symptom of depression and at worst a maintaining cause. If depression evolved because it motivates efforts to solve complex (often social) problems and rumination (careful deliberation) is the means by which it achieves that goal, how is it that the beliefs that people hold when depressed seem to be incorrect (at least to their therapist). We think that there are several possible resolutions to this conundrum.

### Intraspecific Competition Occurs in All Species

First, maladaptive mistakes and failures are an integral part of the human condition. Within every species, individuals compete for scarce resources that are important for survival and reproduction (e.g., food, territories, mates). As a result of that competition, it is inevitable that there are winners and losers. Human beings compete for these resources through situationally dependent cognition and behavior (95). For humans, the social world is incredibly complex and constantly in flux, such that the best strategy often changes from one situation to another. As a result, humans have evolved the cognitive capacity to develop *mental models* of human nature in order to predict how best to behave and what to expect from others in response. Due to differences in genes and experience, some people will develop mental models that work relatively well, while others will develop mental models that work more poorly. In other words, we do not need to invoke the concept of a mental disorder to understand why people develop inaccurate beliefs about their social world. It is simply a necessary consequence of the fact that humans compete to develop better mental models of human nature, and some people are less successful than others in this competition.

But this perspective also suggests that natural selection might have favored the evolution of psychological mechanisms that adjust mental models when they fail to function properly. Mental models are not necessarily “maladaptive” just because they are inaccurate; they are maladaptive if they lead to losses or failures to achieve the resources that make reproduction possible (e.g., mates, food, status, social support). Thus, we argue that the reason why depression is often associated with failures and losses in important domains (e.g., romantic relationships) is because these events suggest that one's mental models of the social world are not working well and need to be revised through the employment of careful methodical Type 2 thinking.

### Evolutionary Mismatch

Second, it is possible that what is going on reflects nothing more than ***evolutionary*** ***mismatch***. Evolved adaptations are traits that exist now because they were shaped by selective pressures that operated in the past (96). Modern environments may deviate substantially from ancestral ones. If so, then what was adaptive in the past may not be adaptive in the present. Most people crave foods that taste sweet. That was adaptive in our evolutionary past when the primary source of simple carbohydrates were fruits that were also rich in vitamins but serves us less well with the advent of processed sugars that lead to obesity and tooth decay. Similarly, starvation was a recurring risk in our ancestral past leading to a preference for the kinds of high caloric foods that raise the risk for metabolic syndrome for those members of the species who have access to an ample supply of meats and starches. From an evolutionary perspective, people have evolved to pay undue attention to how they are treated by close relatives (those who share your genes) and especially by their parents. If your parents do not love or invest in you, that does not bode well for your future. Most recurrence-prone patients have stable (albeit latent) self-images at the core of their depressotypic schemas that they are flawed in some fashion (usually unlovable or incompetent) that predate adolescence. In many instances these beliefs stemmed from the belief (accurate or otherwise) that their parents did not value them and in our ancestral past that could prove to be highly problematic. It likely still is true that being valued by one's parents helps one survive one's childhood, but it is less likely that retaining those negative beliefs about oneself into adolescence helps one navigate complex social relationships as adults. Moreover, the “nuclear family” is a rather modern invention. Children raised in hunter-gatherer societies were usually surrounded by “allo (other) mothers” who contribute the care and nurturing of the child. “Parental investment” in our ancestral past was more a matter of “tribal investment” than it is today.

### Adaptive Search Strategies Are Imperfect

Natural selection causes a species to incrementally increase its fitness, but it does so without foresight or purpose, and it does not guarantee perfection. As Tooby and Cosmides opined “there is no such thing as an adaptation that can maximize fitness under all possible circumstances” (96). The human eye is a good example. It is one of 40 different kinds of “eyes” that evolved in the animal kingdom to process electromagnetic radiation and it functions to let organisms “see” objects at a distance. The human eye contains a “blind spot” at the back of the retina where the optic nerve exits on its way to the brain. No “intelligent designer” would have “designed” an eye that functioned in that fashion (there is nothing adaptive about having a “blind spot” in the back of one's eye and not all species have one) but natural selection does not double back on itself. If a feature represents an improvement over what came before then it tends to be selected regardless of whether some other solution might have worked better. Search-based optimization techniques are useful and often find a superior solution but that does not guarantee that the optimal solution will be found.

The ARH suggests only that people who are depressed will use a slow deliberate “Type 2” processing style to search for a solution to their problems, not that they will always succeed when they do so. It is quite possible that some will get “stuck” for a period of time at a suboptimal solution. Based on clinical (and personal) experience we suspect that it can be quite useful to carefully examine one's own role when things go wrong since that is the easiest thing to correct in the future, but ascribing blame in the form of a stable trait (unlovable or incompetent) is more likely to keep one “stuck” than focusing on the behaviors that one did (or did not) engage in. Traits are simply harder to correct than actions (20). Clinical experience also suggests that those trait ascriptions are more “conditional” than stable and thus still amenable to change. As previously described, much of what gets done in CBT is focused around getting patients to consider alternative explanations for their problems and to examine the existing evidence for each and to run behavioral experiments to test between those competing beliefs. For example, in the case of the sculptor, it was breaking big tasks down into their component parts and doing them one at a time (graded task assignment) that helped him past his tendency to get so overwhelmed by the magnitude of the task that he did not get started. In effect, gathering evidence and running behavioral experiments allows one to correct misguided assumptions and beliefs (it was not that he was “incompetent” just that he chose the wrong behavioral strategy), and thus correct the residue of unfortunate prior experiences (his belief in his own “incompetence” came from being forced to compete with a younger brother for his father's attention and frequently losing out to a sibling who was more outgoing and more facile). What he learned as a young adolescent was not out-of-line with the competition that he faced and the “failures” he experienced; it just was not all that relevant to the challenges he faced as an adult. That said, depression is needed to motivate one to search for the solution to a problem and without that search there is no solution (21).

### Normal Anxiety Can Disrupt Rumination

Getting “unstuck” from a suboptimal solution may involve doing something different than what one has done in the past and for many people that can involve the perception of risk and its attendant affect anxiety. Anxiety often co-occurs with depression [two-thirds of the patients who met criteria for MDD in the DeRubeis and colleagues in the 2005 Penn-Vandy study also met criteria for one or more anxiety “disorder” (69)] but its effect on cognition is different (42).

Whereas, depression leads the individual to ask, “where did I go wrong” and to carefully weigh paths forward, anxiety tends to promote a “better-safe-than-sorry” approach that is often an adaptive response to an imminently dangerous situation (24, 42). Expressing a romantic interest in someone opens one to the risk of rejection and pursing a goal in an achievement domain leaves one at risk for failure, but neither takes one out of the gene pool. Choosing not to act on either does nothing to further the propagation of one's genes.

Earlier we described a teacher who thought that a prior sexual assault as an adolescent undercut her value as a prospective mate and relied on dissimulations and manipulations as compensatory strategies (lying about her past and manipulating romantic partners to get what she wanted) to generate a series of troubled and transitory relationships when in fact it was these interpersonal “safety behaviors” that sabotaged the relationships she formed (20). It was not until she took the chance of leveling with a new romantic partner about what had happened to her in the past (something that took great courage on her part) that she learned that he was not the least concerned about what that meant about her (other than he was sorry that she had been assaulted) and that she could drop the safety behaviors (the lies and manipulations) and simply ask for what she wanted from him in the relationship. Fifteen years she had been stuck on a suboptimal peak because of the anxiety that the thought of full disclosure caused her. The process of climbing down off that suboptimal peak was fraught with a sense of dread that took several months in therapy (and a conversation with a girlfriend and an anonymous survey of “eligible” males) to overcome but the outcome was quite gratifying to her, and she got better (and more comfortable) engaging in self-revelation (as needed) across a series of increasingly satisfying relationships.

### Large Fitness Consequences Can Favor Seemingly Unproductive Cognitions

There is nothing so universally depressogenic as the loss of a child. It is not uncommon for parents who have lost a child to ruminate intensely over what they might have done to prevent the child's death even when it seems clear to others (including the therapist) that there was nothing else they could have done. That being said, understanding the causes of a negative event (even one that has already occurred) can be useful in preventing similar negative events in the future (16, 97).

In our ancestral past, women had an average of about six children over their lifetimes of whom several died (98). Effort spent on understanding the causes of one child's death might help prevent the death of another (99, 100). Watching parents engage in self-recriminating rumination might seem cruel, but the fitness costs are so great that natural selection would have favored the expenditure of a great deal of cognitive effort even if it only had a miniscule chance of increasing the odds of survival for the other children. We focused on the loss of a child in this example, but the same principle extends to any situation in which the fitness consequences are great.

As Dawkins describes in his 1976 treatise “*The Selfish Gene*,” we are but “survival machines” engineered by natural selection to propagate our gene lines at all times even if at our own affective expense (101). An evolutionary perspective would suggest that there is little point in trying to convince grieving parents not to engage in a causal analysis in such a situation (or other patients from grieving in the aftermath of a romantic breakup or the loss of a job) but rather to point out that the brain is designed to explore the possible causes of negative life events on the off chance that such events can be prevented in the future. To ruminate in response to loss or failure is an eminently “species-typical” (human) thing to do. The optimal response in CBT is to label it as an attempt to solve a problem (or prevent a future one) and to help the process along.

### Inclusive Fitness Theory

As previously noted, one of the most important insights in evolutionary biology over the last century is that organisms are not designed by natural selection to maximize their own survival or even their own reproductive success but rather to maximize the reproductive success of their gene line (102). This is what Dawkins meant when he labeled us as nothing more than “survival machines” (101). Individuals not only propagate their gene lines through their own reproductive efforts (***direct fitness***) but also via propagating the reproductive success of their biological relatives (***indirect fitness***). The sum of direct and indirect fitness is called ***inclusive fitness*** (103), and it is this sum that best predicts of what kinds of behaviors organisms engage in because that is what is actually maximized by natural selection (102).

The essence of the idea was captured by the iconic quip by the evolutionary geneticist J. B. S. Haldane who was reported to have said that he would not sacrifice his life for his brother, but he would do so for two brothers or eight cousins (104). This phenomenon is easiest to see in the lives of social insects. Only a small percentage of the individuals actually reproduce (the queen and one or more of the male drones) while the vast majority labor to ensure the propagation of a gene line comprised solely of their biological siblings. This concept is crucial in explaining many important biological events including multicellularity, apoptosis and other forms of programmed cell death, as well as the evolution of social systems characterized by family groups and parenting behavior in humans. Where it intersects especially with clinical concerns has to do with self-sacrifice. No one would question a parent's willingness to sacrifice his or her life for the life of his or her child, but not all would see the same genetic mechanism “baked in” to the suicidal ruminations of a person who is concerned about being a burden to biological relatives.

In not-so-distant times amongst peoples who lived on the edge starvation in northern climes (like the Inuit north of the Arctic circle), it would be considered “de rigueur” for post-reproductive elders to walk out into the snow and not come back if the winters were too long and their grandchildren faced starvation as a consequence (105). Such “altruistic” notions might seem misguided in situations in which starvation is not imminent (suicide is the “gift that keeps on giving” to the survivors) but the psychological mechanism would have been selected for in our ancestral past in a manner wholly in keeping with the concept of inclusive fitness.

Many people who die by suicide believe that their families would be better off without them (106). Most patients entertain at least “passive” suicidal ideation, and over half of all people who die by suicide have a history of depression. Self-sacrificial impulses would be favored by natural selection among those individuals who see themselves as defective or impaired and those with a history of childhood abuse (self-esteem is often based on parent's behavior). People with a history of failed relationships also are at risk even during the reproductive years (107–109).

If some of our readers have a visceral response to the use of the word “adaptive” to describe suicide and other forms of self-destructive behavior, this is an indication that the evolutionary perspective is novel and non-intuitive. ***Clinicians need to understand the*** ***naturalistic fallacy*. ***An ‘is' is not an ‘ought.' Cancer ‘is' a collection of cells that are pursuing their inclusive fitness. It is hardly an “ought,” but intervention ‘is' nevertheless warranted*. Moreover, we should not let moral repugnance bias the scientific study of human behavior. Prolicide (killing one's offspring), the killing of conspecifics, and sexual coercion are common throughout the animal kingdom, and humans are no different. We strongly advocate for clinical intervention in situations in which people are engaging in self-destructive behavior as part of the pursuit of indirect fitness interests. We also think that it is likely to help the patient to identify the evolutionary origins of seemingly maladaptive behaviors, such as rumination and suicide. Not all evolved adaptations need to be implemented if they are not consistent with the patient's current interests (most reproductively capable adults practice birth control from time-to-time). Making treatment more efficacious will require differentiating psychological phenomena that result from some malfunction in the brain from those mechanisms that evolved to maximize inclusive fitness. Any effective and efficient treatment must fit an accurate model of human nature and depression.

## Summary And Conclusions

An adaptationist evolutionary theory suggests that depression is an adaptation that evolved because it increases inclusive fitness in response to negative life events and the ARH suggests that it does so by increasing the propensity to ruminate. Whereas, most clinicians see rumination as a symptom of depression, or even worse, a cause, the ARH sees it as a means to move from a careful causal analysis of a complex social problem to a workable solution. Most episodes of depression remit on their own in the absence of treatment, suggesting that whatever adaptation evolved in our ancestral past works well in most instances. CBT is efficacious in the treatment of depression and has an enduring effect that reduces future risk but may only be needed for those among the “recurrence prone” who get “stuck” temporarily making internal stable attributions that provide no clear behavioral path to resolution. ADMs are analgesic at best in that they treat the symptoms but not the problem, and quite possibly iatrogenic with respect to prolonging the underlying episode and worse creating harmful dysfunctions in other areas of the body and brain. CBT might be preferred over ADMs if it facilitates the functions that depression evolved to serve.

## Data Availability Statement

The original contributions presented in the study are included in the article/supplementary material, further inquiries can be directed to the corresponding author.

## Author Contributions

All authors listed have made a substantial, direct and intellectual contribution to the work, and approved it for publication.

## Conflict of Interest

The authors declare that the research was conducted in the absence of any commercial or financial relationships that could be construed as a potential conflict of interest.
